# Intestinal nontuberculous mycobacteria infection: A case report

**DOI:** 10.1097/MD.0000000000036954

**Published:** 2024-02-16

**Authors:** Yanbin Xu, Jinfeng Yang, Lili Cui, Chengchen Huang, Chun Wu

**Affiliations:** a Department of Multidrug Resistant Tuberculosis, Changchun Infectious Disease Hospital, Changchun, China; b Department of Medical Affairs, Shanghai Conlight Medical Laboratory Co., Ltd, Shanghai, China.

**Keywords:** case report, colonoscopy biopsy, intestinal nontuberculous mycobacteriosis, nontuberculous mycobacteria, nucleotide-based MALDI-TOF MS

## Abstract

**Background:**

Intestinal nontuberculous mycobacteriosis due to nontuberculous mycobacteria infection has clinical manifestations similar to intestinal tuberculosis and inflammatory bowel disease, causing difficulties in clinical diagnosis.

**Case presentation:**

A 42-year-old male patient was admitted to the Sino-Japanese Friendship Hospital of Jilin University in June 2021 for diarrhea and intermittent hematochezia since April 2021. He was diagnosed with inflammatory intestinal disease by colonoscopy and midtransverse colon biopsy. However, the symptoms did not relieve after 2 months of mesalazine treatment. In August 2021, the patient was admitted to the outpatient department for suspected “intestinal tuberculosis.” A diagnosis of intestinal nontuberculous mycobacteriosis was confirmed based on pathology and nucleotide-based matrix-assisted laser desorption/ionization time of flight mass spectrometry (MALDI-TOF MS). After 2 weeks of antimycobacterial therapy, the patient’s diarrhea was relieved, and hematochezia no longer appeared. In November 2021, recolonoscopy revealed scattered erosions and ulcers in ileocecal valve and ascending colon, while both nucleotide-based MALDI-TOF MS and next-generation sequencing could still detect *Mycobacterium intracellulare*.

**Conclusion:**

This study reported a patient with an intestinal nontuberculous mycobacteriosis diagnosed by colonoscopy biopsy and nucleotide-based MALDI-TOF MS, and symptoms were relieved after antimycobacterial treatment.

## 1. Introduction

Nontuberculous mycobacteria (NTM) refer to all mycobacteria except *Mycobacterium tuberculosis* complex and *Mycobacterium leprae*.^[1]^ More than 190 species and 14 subspecies of NTM have been found, most of which are parasitic bacteria.^[2]^ A small portion of them is pathogenic to the human body and belongs to an opportunistic pathogen.^[3]^ Intestinal nontuberculous mycobacteriosis has similar clinical manifestations to intestinal tuberculosis and inflammatory intestinal disease, leading to the difficulty of diagnosis, frequent misdiagnosis, and mistreatment in the clinic consequently.^[4]^

Matrix-assisted laser desorption/ionization time of flight mass spectrometry (MALDI-TOF MS), with its advantages of user friendliness, simple workflow, short turn-around time, and low cost, has been widely used in the analysis of nucleotide, proteins, and other biological macromolecules,^[5]^ promising to be extended in clinic. Based on this technique and taking nucleotides from specimens as objects, a commercial product, Conlight Myco Assay, is also available to detect mycobacterial species accurately and rapidly.

Here, we reported a case of intestinal nontuberculous mycobacteriosis (*Mycobacterium intracellulare*) diagnosed by colonoscopy biopsy and nucleotide-based MALDI-TOF MS.

## 2. Case presentation

**Figure 1.**
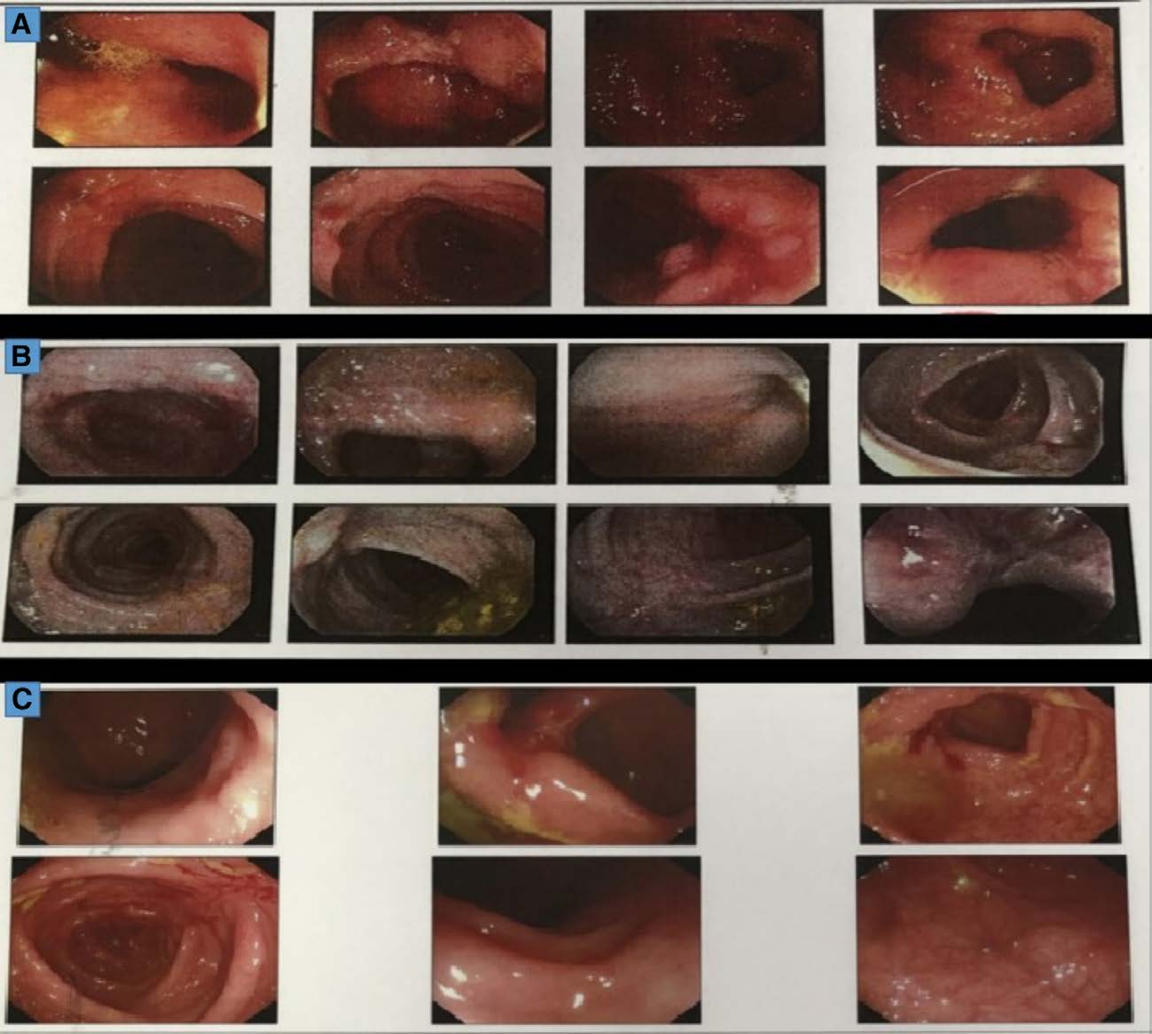
Colonoscopy result of the patient. (A) The first colonoscopy on June 2021, showed large and deep ulcers distributed in stages within the visible range from the cecum to the colon. (B) The second colonoscopy on August 2021 showed multiple lamellar ulcers at the end of the ileum with a diameter of 0.2–0.6 cm, and many flaky hyperemia spots were covered with white fur. (C) The follow-up colonoscopy on November 2021, showed scattered punctate erosions in the terminal ileum without abnormality in the opening of the appendix, scattered erosions, and ulcers in the ileocecal valve and ascending colon, scattered congestion, and digital polyps in the transverse colon and descending colon.

A 42-years-old male patient was admitted to the Sino-Japanese Friendship Hospital affiliated to Jilin University on June 2, 2021, for diarrhea and intermittent hematochezia since April 2021. Colonoscopy showed large and deep ulcers distributed from the cecum to the colon, about 30-cm away from the anal verge. Some ulcers had paving stone-like changes around the ulcer, the blood vessels were blurred, the ileocecal valve was damaged, and the terminal ileum showed hyperemia and edema (Fig. 1A). Two ulcer biopsies showed acute and chronic mucosa inflammation with necrosis and inflammatory granulation, local crypt abscesses, abundant lymphocytes, and lobulated nuclear cells in the interstitium. The patient was diagnosed with “inflammatory intestinal disease” and treated with mesalazine for 2 months, without relief.

On August 3, 2021, a reexamination of colonoscopy showed multiple lamellar ulcers at the end of the ileum with a diameter of 0.2–0.6 cm, and many flaky hyperemia spots were covered with white fur. The ileocecal valve structure was damaged and deformed, the appendix opening was normal, and flaky hyperemia spots could be seen in the cecum. The segmental distribution of deep ulcers could be seen in the ileocecal valve, from the ascending colon to about 40-cm away from the anal verge. Some ulcers showed paving stone-like changes (Fig. 1B).

Two ulcer biopsies near the ileocecal and ascending colon showed acute and chronic mucosal inflammation with necrosis and inflammatory exudation. The symptoms and intestinal lesions did not relieve after treatment. The patient was admitted to the outpatient department on August 10, 2021, for suspected “intestinal tuberculosis.” Abdominal CT showed a blurred intestinal wall around the ileocecal region and the surrounding fat space. Protein chip tuberculosis antibodies of 38 kD, 16 kD, and LAM were negative. Tuberculin skin test (PPD) was 0*0, and the T-cell spot test (T-SPOT.TB) was negative. A colonoscopy was performed 3 d later, and the results were roughly the same as before. Nucleotide-based MALDI-TOF MS on biopsies (5 pieces of ulcers in the terminal ileum, ileocecal, cecum, and ascending colon) found *Mycobacterium intracellular* and *Mycobacterium nonchromogenicum*. The diagnosis was intestinal nontuberculous mycobacteriosis (*Mycobacterium intracellulare*). Then the patient was admitted to the hospital for treatment.

Antimycobacterial therapy was used with clarithromycin, rifabutin, amikacin, linezolid, and clofazimine. The drug dose was adjusted according to the patient’s weight. After 2 weeks of antimycobacterial therapy, the patient’s diarrhea was relieved, and hematochezia no longer appeared. After 2 months of treatment (the treatment was interrupted for 1 month due to the patient’s adverse drug reactions), colonoscopy in November 2021 showed scattered punctate erosions in the terminal ileum without abnormality in the opening of the appendix, scattered erosions and ulcers in the ileocecal valve and ascending colon, scattered congestion, and digital polyps in the transverse colon and descending colon (Fig. 1C). A total of 8 biopsies were collected, both nucleotide-based MALDI-TOF MS and next-generation sequencing could still detect *Mycobacterium intracellulare*. The patient continued with antimycobacterial treatment for now.

## 3. Discussion

Nontuberculous mycobacteriosis caused by *Mycobacterium intracellulare* is often clinically relevant.^[6–10]^ A recent case reported that a diffuse NTM disease caused by *Mycobacterium intracellulare* occurred in a child with IFNGR1 deficiency, with a good prognosis after antimycobacterial treatment.^[11]^ A patient diagnosed with septic arthritis was due to NTM infection as *Mycobacterium intracellulare* was isolated by culture and showed an excellent prognosis with antimycobacterial treatment.^[12]^

This study shows that the clinical application of molecular diagnostic technology plays a crucial role in diagnosing nontuberculous mycobacteriosis and the differential diagnosis with other inflammatory disorders.^[13]^ The method has a specificity of 98.6% and takes about 1–2 hours to obtain results and has been widely used in clinical practice.^[14]^ *Mycobacteria* were identified to the species level by analyzing different characteristic protein profiles with various cytoplasmic/nucleus ratio protein components during vacuum ionization. Furthermore, nucleotide-based MALDI-TOF MS was developed to identify numerous mycobacterial species, including MTBC and NTM^[15]^ as well as their drug-resistance spectrum^[16]^ with high sensitivity and specificity.

Next-generation sequencing can also be used to track the spread of NTM in specific populations.^[17,18]^ In this case, the results of the above 2 molecular diagnostic techniques were consistent, showing that the diagnostic results were credible. In addition, collecting pathological tissues during endoscopy for the diagnosis is also the key to improving diagnosis accuracy.

Antimycobacterial therapy adopted in this study was supported by published evidence. Clarithromycin, rifabutin, and amikacin are considered empiric therapy for slowing growing NTM (i.e., *Mycobacterium intracellulare*), while linezolid is a second-line agent and clofazimine is a new antimicrobial for treatment of NTM.^[19,20]^ The optimal drug combinations have not been reliably established for NTM infections, and the clinical decision is often based on clinical experience and expert opinion.^[20]^

In conclusion, this study reported a patient with an intestinal nontuberculous mycobacteriosis diagnosed by colonoscopy biopsy and nucleotide-based MALDI-TOF MS assay, and symptoms were relieved after antimycobacterial treatment.

## Acknowledgments

We appreciate the case for the information consent for this study.

## Author contributions

**Conceptualization:** Chun Wu.

**Data curation:** Jinfeng Yang.

**Formal analysis:** Yanbin Xu, Lili Cui.

**Funding acquisition:** Chun Wu, Chengchen Huang.

**Investigation:** Yanbin Xu, Lili Cui.

**Methodology:** Yanbin Xu, Chengchen Huang.

**Project administration:** Chun Wu.

**Resources:** Chun Wu, Chengchen Huang.

**Software:** Yanbin Xu.

**Supervision:** Chun Wu, Chengchen Huang.

**Visualization:** Yanbin Xu.

**Writing – original draft:** Yanbin Xu.

**Writing – review & editing:** Chun Wu, Chengchen Huang.
